# EPD1504: a novel μ-opioid receptor partial agonist attenuates obsessive–compulsive disorder (OCD)-like behaviors

**DOI:** 10.3389/fpsyt.2023.1170541

**Published:** 2023-06-30

**Authors:** Beth Youngblood, Julio C. Medina, Donald R. Gehlert, Neil Schwartz

**Affiliations:** ^1^Epiodyne, Inc., San Francisco, CA, United States; ^2^R2M Pharma Inc., South San Francisco, CA, United States

**Keywords:** treatment resistant OCD, opioid, buprenorphine (BN), probabilistic reversal task, marble burying (MB), partial agonist, obsessive–compulsive disorder (OCD)

## Abstract

Low doses of μ-opioid receptor (MOR) agonists rapidly ameliorate symptoms in treatment-resistant obsessive–compulsive disorder (OCD) patients (10–50% of OCD patients). However, the utility of MOR agonists is limited by their safety liabilities. We developed a novel MOR partial agonist (EPD1540) that has an improved respiratory safety profile when compared to buprenorphine. Buprenorphine is a MOR partial agonist primarily used in the treatment of opiate-use disorder, which in investigator-led trials, has been shown to rapidly ameliorate symptoms in treatment-resistant OCD patients. In this study, we show that doses of EPD1504 and buprenorphine that occupy small fractions of MORs in the CNS (approximately 20%) are as effective as fluoxetine at ameliorating OCD-like behaviors in two different rat models (an operant probabilistic reversal task and marble burying). Importantly, effective doses of EPD1504 did not impair either locomotor activity, or respiration under normoxic or hypercapnic conditions. Additionally, EPD1504 had effects comparable to buprenorphine in the conditioned place preference assay. These results indicate that EPD1504 may provide a safer alternative to buprenorphine for the treatment of OCD patients.

## 1. Introduction

Approximately 50% of obsessive–compulsive disorder (OCD) patients do not respond to first-line serotonergic treatments (1–3). In these treatment-resistant patients, μ-opioid receptor (MOR) agonists ameliorate OCD symptoms, whereas in many cases, MOR antagonists exacerbate OCD symptoms (4–11).

Several experimental approaches have been developed to investigate circuit and behavioral mechanisms underlying OCD (12, 13). These tasks measure behavioral flexibility in both humans and rodents; examples include the probabilistic reversal task that is disrupted in OCD patients (14), and that is modulated by manipulations targeting serotonin receptors in both healthy volunteers (15, 16) and rodents (17, 18). Another widely used model of compulsions associated with OCD is marble burying in rodents; in this assay, multiple classes of central nervous system (CNS)-active drugs modulate the rodents’ innate behavior to bury marbles placed in their cage (19–23).

Although both morphine and tramadol (a prodrug with opioidergic and monoaminergic polypharmacology) ameliorate OCD symptoms in treatment-resistant patients (6, 8, 10), their utility is limited due to inherent respiratory and abuse liabilities (24); in the case of tramadol additional limitations include variable metabolism (conversion from prodrug to active form), nausea, and increased risk of seizures (25, 26). Compared with both morphine and the metabolites of tramadol that bind the MOR, the partial MOR agonist buprenorphine has lower intrinsic MOR efficacy and significantly higher MOR affinity (27-29). These properties appear to limit its respiratory depressant and associated euphoric effects. Because of its better safety profile and high affinity for the MOR, buprenorphine is primarily used as a maintenance therapy to both relieve cravings for, and to occlude the euphoric and respiratory depressant effects of opioids of abuse (30). Not surprisingly, in small investigator-led clinical trials buprenorphine has been shown to ameliorate OCD symptoms and has a slightly better safety profile than the higher efficacy agonists such as morphine and tramadol (4, 9). However, its utility is limited due to variable pharmacokinetics, including a series of metabolites that are high-efficacy MOR agonists (31, 32), and respiratory depression that cannot be rescued by the standard of care (standard clinical doses of the MOR antagonist naloxone) (33). Furthermore, because buprenorphine is primarily used in the treatment of opioid abuse, access to buprenorphine is highly regulated, e.g., in 2016, buprenorphine was removed from the morphine milligram equivalence (MME) scale—a scale used to standardize opioid prescriptions (30, 34).

Based on the clinical, regulatory, and preclinical evidence, a MOR partial agonist with buprenorphine-like efficacy and reduced respiratory liabilities would provide a safer and more accessible alternative for treatment-resistant OCD patients. Therefore, we designed and tested the MOR partial agonist EPD1504—a MOR partial agonist with buprenorphine-like intrinsic MOR efficacy. Our previous study reported on RM1490 a molecule with significantly lower intrinsic MOR efficacy (35). In preclinical rat models, EPD1504 was compared to both serotonin (hydroxytryptamine [5HT]) and MOR ligands in two models of OCD-like behavior (probabilistic reversal and marble burying assays). Serotoninergic ligands included fluoxetine, a 5HT reuptake inhibitor— that has been shown to ameliorate OCD symptoms and to reduce marble burying (20), and mCPP, a serotonin receptor and transporter ligand, —that depending on on dose, species, and experimental setup, has been shown to both exacerbate and attenuate OCD patient symptoms and rodent marble burying (20); mCPP was, therefore, used a positive control in the probabilistic reversal task. The MOR ligands included the partial MOR agonist buprenorphine and the MOR antagonist naloxone.

## 2. Methods

### 2.1. Compounds

**Figure 1:**
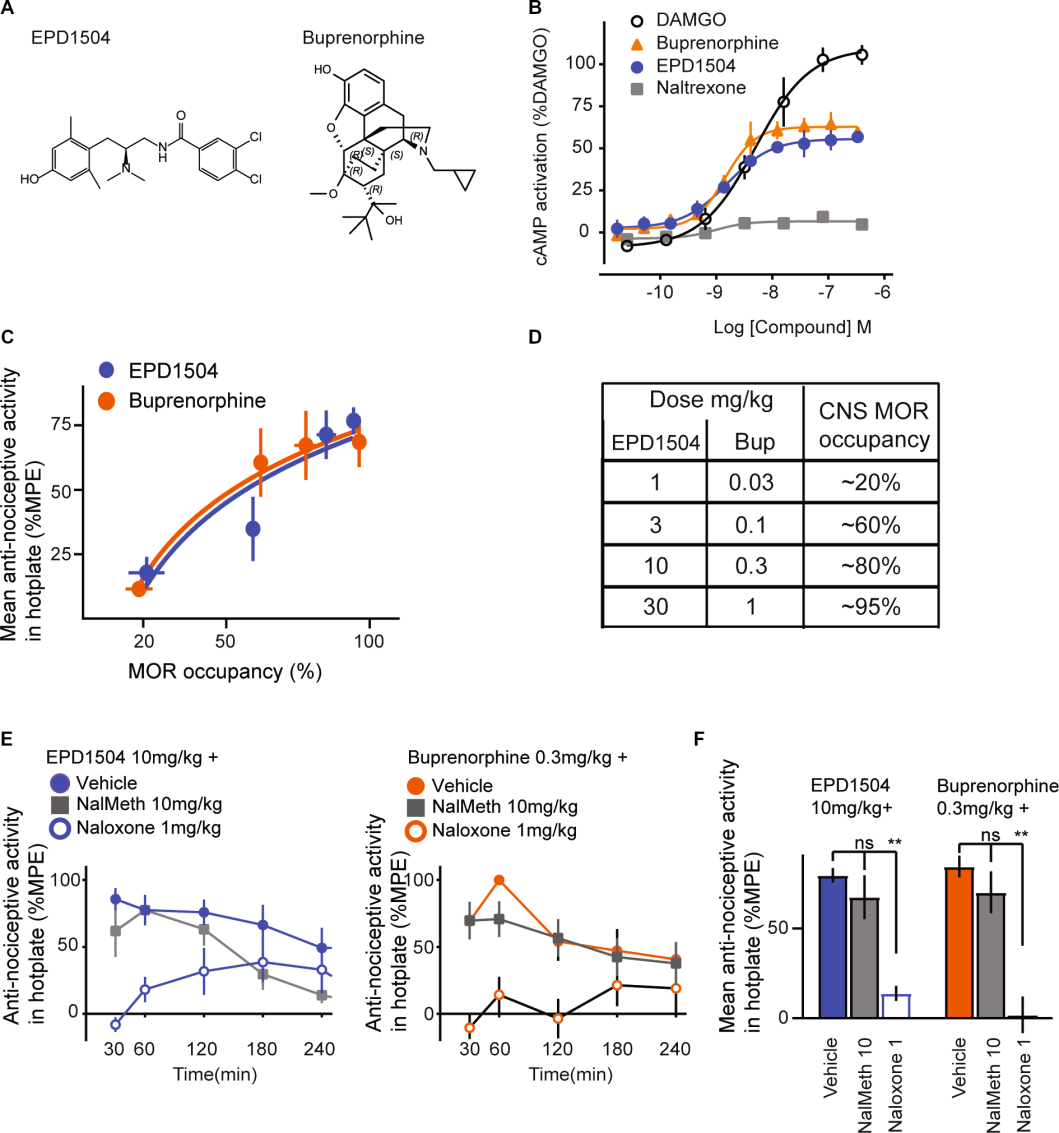
EPD1504 is a brain penetrant MOR partial agonist with buprenorphine-like antinociceptive activity in the hotplate test. **(A)** Chemical structures of EPD1504 and buprenorphine. **(B)** Representative dose–response curves for cAMP activation in CHO cells expressing human MORs, with EPD1504, the full agonist: DAMGO, antagonist: naltrexone, and comparable partial agonist: buprenorphine (*n* = 3 replicates per experiment, mean ± SD). **(C)** Coplot of dose–response curves for antinociceptive activity in the hotplate test measured as the mean of % maximum possible effect (%MPE) between 30 and 120 min after injection of the test compound on the *y*-axis vs. %MOR occupancy in the thalamus at 60 min after injection of the test compound on the *x*-axis (rats per dose: %MOR occupancy, *n* = 3–4, %MPE antinociceptive activity, *n* = 6–8). **(D)** Summary table for dose and percent occupancy of MOR for the data shown in **(C)**. The data used to calculate CNS MOR percent occupancy for buprenorphine have been reported in our previous study (35). **(E)** Time course and summary data is shown in **(F)** of antinociceptive activity in the hotplate test for doses of EPD1504 10 mg/kg and buprenorphine that occupy approximately 80% of MOR in the thalamus; note that the antinociceptive activity of both agonists was antagonized by naloxone (1 mg/kg) (which crosses the blood–brain barrier), but not by naloxone methiodide 10 mg/kg which does not cross the blood–brain barrier (*n* = 6–8 rats per dose, ANOVA, **p < 0.01, ns = not significant).

Naloxone, naltrexone, buprenorphine hydrochloride, DAMGO, forskolin, IBMX, naloxone hydrochloride, fentanyl, and diazepam were obtained from Sigma–Aldrich, St. Louis, MO, USA. Carfentanil was obtained from Cayman Chemical, Ann Arbor, MI, USA. C Ham-F12 and FBS were purchased from Invitrogen, Carlsbad, CA, USA. EPD1504 was provided by R2M Pharma, South San Francisco, CA, USA and synthesized as reported in our previous study (35); a schematic of EPD1504 is shown in Figure 1A. Naloxone, naltrexone, and fentanyl were dissolved in 0.9% saline; stock solutions of the other compounds were prepared by dissolving in DMSO and kolliphor and then diluting with 0.9% saline to working concentrations, which contained 1% or less of DMSO and kolliphor; vehicle control solutions consisted of 0.9% saline, 1% DMSO, and 1% kolliphor. Unless otherwise stated, injection was subcutaneous (s.c.) at 3–5 mL/kg. ALZET minipumps (2ML1: flow rate 10 μL/h for 7 days) purchased from Durect Corporation, Cupertino, California was used for subchronic dosing.

### 2.2. Cell culture and *in vitro* assays

Experiments were performed at R2M Pharma, South San Francisco, CA, USA. MOR cAMP assays: CHO TAG-LITE human MOR stable cell line from Cisbio (NCBI accession No. NM_000914.3, Bedford, MA, USA) were seeded and grown to approximately 80% confluence in Ham F-12 with 10% fetal bovine serum (FBS), 50 U/mL penicillin, 50 μg/mL streptomycin, 2 mM Hepes, and 1 mg/mL geneticin (Invitrogen, Carlsbad, CA, USA). Cells were then harvested using Accutase (Corning, Corning, NY, USA), centrifuged at 1,300 *g* for 5 min, and plated at 5,000 cells per 5 μL/well in a 5× dilution of stimulation buffer consisting of the HTRF cAMP G_i_ kit, water, and IBMX at 0.5 mM (Cisbio, Bedford, MA, USA) in white HTRF low volume 384-well plates (Cisbio, Bedford, MA). Plates were then incubated at 37°C in 5% CO_2_ for 10 min. For the agonist assay, forskolin was added to a final concentration of 4 μM. For the antagonist assay, forskolin (4 μM) and DAMGO at EC_90_ final concentration were added. Test compounds were dissolved in DMSO and water, then serially diluted to working concentrations such that the concentration of DMSO was less than 0.1%. Diluted test compounds were added at 2.5 μL/well; plates were incubated at 37°C and 5% CO_2_ for 15 min and then at room temperature for 15 min. Next, 5 μL/well of cAMP Eu-cryptate and 5 μL/well of anti-cAMP-d2 (both diluted 1:20 in lysis buffer) were added and plates were incubated at room temperature for 1 h. Following incubation, plates were read in a Synergy Neo2 multimode reader (Biotek, Winooski, VT). Plate reader settings were set to homogeneous time-resolved fluorescence (HTRF) with excitation at 330 nm and emissions of 620 and 665 nm. Emission fluorescence was normalized (665/620 nm signal × 1,000). For the agonist assay, data were normalized using the maximal DAMGO response. The measurements were performed in triplicate, and the dose–response curves were fit using non-linear regression.

### 2.3. Animals

Male Sprague Dawley rats (225–300 g) from Charles River were used for all experiments. Unless otherwise stated, animals were acclimatized to the local holding facility for at least 3 days after arrival from Charles River. Animals were maintained on a 12-h light cycle. The experiments involving animals were approved by the Life Source Biomedical Services Institutional Animal Care and Use Committee (IACUC) in accordance with the animal care standards set forth by the Office of Laboratory Animal Welfare (OLAW), the National Institutes of Health (NIH).

### 2.4. Hot plate

Equipment and procedures were based on the methods discussed in our previous study (35). After 2 days of acclimatization, hotplates were switched on and set to 52°C. To determine the baseline, vehicle was injected, and 15 min later, the animal was placed on the hotplate. An experimenter, blinded to the treatment condition, monitored the animal for responses including a lick of a hind paw, shake of a hind paw, or stomping of the hind paws; after either two of the listed observations, or if the animal vocalized, jumped (all 4 feet off the ground), or reached the nominal cutoff time of 30 s without exhibiting a detectable response, the animal was immediately removed from the hotplate, returned to its home cage, and the time was recorded. To determine the test score, on the next day, the procedure was repeated following the injection of the test compound. If an animal did not respond to touch upon being picked up for testing, its righting reflex was tested; animals that did not right in 5 s were assigned the cutoff score of 30 s, and were not tested at that time point. Percentage maximum possible effect (%MPE) was calculated as 100*[(test − baseline)/(30 s − baseline)], where 30 s is the cutoff time. The average effect in summary bar graphs was calculated as the average test scores between 30 and 120 min.

### 2.5. Receptor occupancy

Experiments were performed at Melior, Inc. procedures and data used for buprenorphine were reported in our previous study (35). Concisely, test compounds were injected subcutaneously (s.c.), and 40 min later, the tracer (carfentanil) was injected intravenously (i.v.) through a lateral tail vein. After 20 min, animals were sacrificed, and cerebellar and thalamic tissue samples were collected and processed for liquid-chromatography tandem mass spectrometry. To calculate occupancy, the concentration of carfentanil detected in the thalamus (MOR-containing region) was normalized to the levels in the cerebellum (MOR-null region).

### 2.6. Probabilistic reversal: training

**Figure 2:**
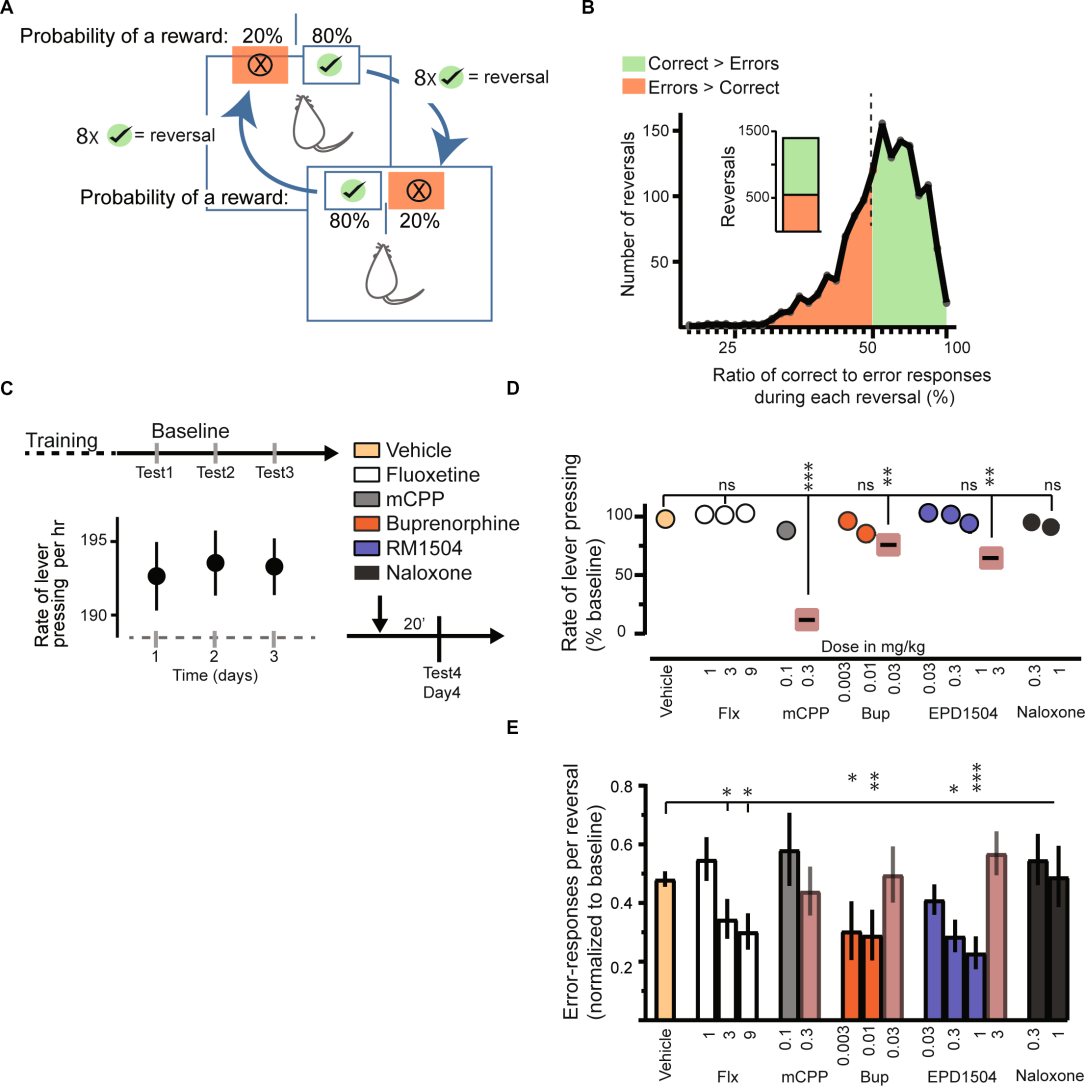
EPD1504 and buprenorphine reduce error responses in a probabilistic reversal task. **(A)** Schematic of the probabilistic reversal task. A food restricted rat can press either lever in the operant chamber to obtain a food reward. The probability of a reward after pressing the “correct lever” (green with check mark) is 80%, whereas the probability of a reward after pressing the “error lever” is 20%. After eight correct-lever presses the contingencies reverse, i.e., the correct lever becomes the error lever and vice versa. **(B)** Histogram of 1,410 reversals during baseline sessions (*n* = 62 rats). Note that rats had learned the contingencies of the task as more correct responses were made during a greater percentage reversal (approximately 60% of reversals). **(C)** Baseline rate of responding for 3 days. **(D)** Rate of responding during subsequent test trials. Note that doses that reduced the response rate were excluded from further analysis. **(E)** Error responses per reversal normalized to the interquartile range of responses during baseline [data in **(B)**]; (ANOVA, *p < 0.05, **p < 0.01, ***p <0.001, ns = not significant; *n* = 3–4 rats per group, *n* = 2 daily sessions).

Equipment and procedures were based on methods discussed in previous studies (17, 18, 36). Training and testing were carried out in custom-built operant chambers fitted with equipment from Med Associates, Inc. (Fairfax, VT, USA), including a house light, fans, a tone generator, and two retractable levers positioned opposite to a food magazine fitted with a photo-beam sensor to detect entries. Concisely, food-restricted rats (approximately 95% free-feeding weight) were first trained to press either lever on an FR1 schedule; each lever press initiated a tone, a retraction of both levers, and the delivery of a 45-mg chocolate food pellet (Bio-Serv, NJ, USA). The tone continued for 10 s or until the reward was collected from the food magazine. The intertrial interval was 15 s. Once rats earned 30 pellets in 1 h, the probability of a reward was reduced to 70% on both levers. Animals showing a bias for one of the levers (>70% of response on one lever only) were excluded. After 2 days, the reward probability on the levers was set to 80% and to 20%, which denoted “correct” and “error” levers, respectively. After eight presses on the “correct” lever, the contingencies were reversed (the 80% lever becomes 20% lever and vice versa). A schematic of the task is presented in Figure 2A.

### 2.7. Probabilistic reversal: testing

After training, baseline was established: animals were injected 20 min before each daily session with the vehicle. Each session was run for approximately 1 h, or until 200 lever presses were completed. Individual baselines were recorded once there were > 6 reversals per hour and < 25% difference between the rate of reversals during three daily sessions. The rats that failed to meet these criteria in eight sessions were excluded. The following day, the rats were injected with test compounds 20 min before a session.

### 2.8. Marble burying

**Figure 3:**
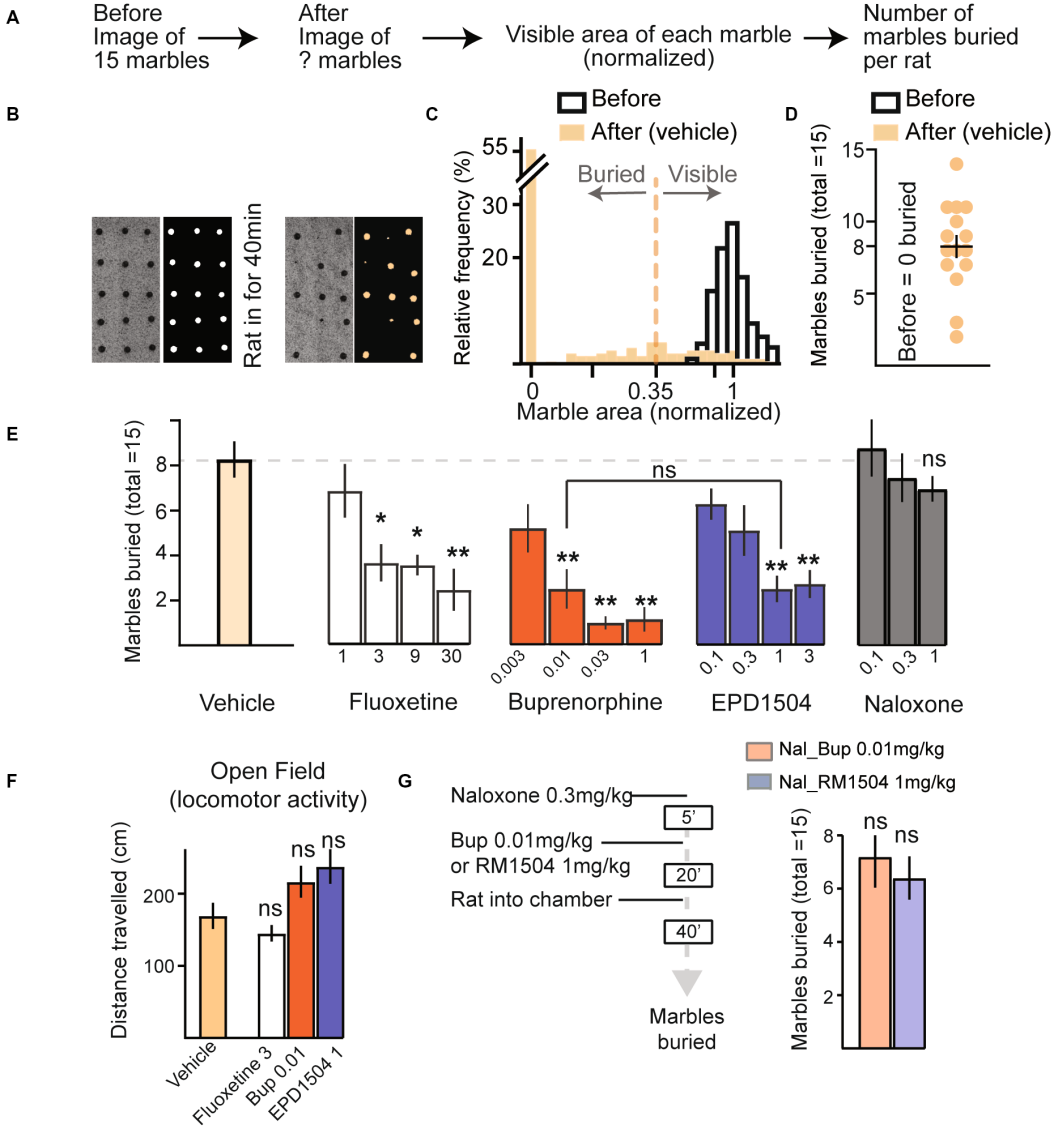
EPD1504 and buprenorphine reduce marble burying. **(A)** Timeline of semi-automated method to quantify marble burying. **(B)** Representative photographs and processed images of marbles on the bedding (*n* = 15 marbles, before the rat was placed in the chamber), and after the rat had been in the chamber for 40 min. **(C)** Histogram showing cutoff used to determine whether a marble is buried or visible for 1,155 marbles before the rat was placed in the chamber (open bars), and after the rat was placed in the chamber (filled bars); note that no marbles were classified as buried before the rat was placed in the chamber. **(D)** Summary data for vehicle treated rats (*n* = 15). **(E)** Mean marbles buried for data in **(D)**, and dose–response curves for marbles buried by rats injected with either vehicle (*n* = 18), fluoxetine (*n* = 10 per dose), buprenorphine (*n* = 10 per dose), EPD1504 (*n* = 11 per dose), or naloxone (*n* = 10 per dose). Doses are listed as mg/kg below the *x*-axis. **(F)** Doses of fluoxetine (3 mg/kg), EPD1504 (1 mg/kg), and buprenorphine (0.01 mg/kg) that reduced the number of marbles buried (did not affect distance traveled in the open-field test, and did not affect rate of lever pressing in the probabilistic reversal task as shown in Figure 2). **(G)** Timeline and data for rats preinjected with naloxone (1 mg/kg) followed by either EPD1504 (1 mg/kg) or buprenorphine (0.01 mg/kg) [*n* = 5 per group, *p<0.05, **p<0.01 ANOVA, not significant vs. vehicle treated rats shown in **(E)**].

Equipment and procedures were based on published methods (20, 21). Clean housing cages were filled with 5 cm of clean home cage bedding. Red glass marbles of 1/2 inch diameter were evenly distributed onto the bedding using a custom-made plexiglass template with 15 holes. Under uniform lighting, a “before” image of the bedding and 15 marbles was taken using a Logitech C920 Webcam. The cage was then placed in a sound-attenuating box purchased from Med Associates, Inc. (Fairfax, VT, USA). The rats were injected with test compounds and after 20 min the animals were placed in the cage (one rat per cage with the marbles). The cage was then covered with its lid. After 40 min, the rats were removed and were returned to their home cage. A second “after” image was then taken of the bedding and marbles. Representative images for “before” and “after” of the bedding along with marbles on it are shown in Figure 3B. Images were analyzed using ImageJ software (NIH). To determine the number of marbles buried, the image on the red channel (color of the marbles) was converted to a binary image, and each marble’s visible area was determined using the analyze particle tool. The visible area of each marble was divided by the mean of the marbles’ areas before rats were placed into the cage with the bedding and marbles. As shown in Figures 3A–D, a marble was considered buried if <0.35 of its area was visible. This cutoff agrees with the standard of two-thirds of buried areas (i.e., <0.33 visible) as estimated by the experimenter in the previous studies (20, 21).

### 2.9. Conditioned place preference (CPP) using opioid–naïve rats

The recording chamber consisted of two webcameras and a custom-built plexiglass chamber 38′′ × 38′′ × 18′′ that was further enclosed in a sound-attenuating box purchased from Med Associates, Inc. (Fairfax, VT, USA). Animals were tracked using the videography from the camera positioned above the chamber using the Viewer2 software (Biobserve GmbH). To determine the baseline time during the pretest, a divider with a cutout door (5′′ × 5′′ × 4′′) was used to divide the chamber into two sides of equal dimensions; each side of the chamber had distinct visual and tactile cues including floors of different consistency (smooth vs. bumpy), visual cues (2 cm stripes, vertical vs. horizontal). The time animals spent freely exploring each side of the chamber was measured for 20 min (1,200 s). To reduce the effect of intrinsic biases, animals that spent >900 s on one side of the chamber were excluded from the study. Conditioning began the following day: s.c. injection of vehicle in the morning (between 8 a.m. and 11 a.m.) and test compound 4 h later in the afternoon (between 12 p.m. and 3 p.m.). After each injection, animals were confined to one side of the chamber for 45 min and then were returned to their home cage. The injections and conditioning procedure were repeated 2× at 48 h intervals. Two days after the last injection, the divider with the cutout was placed in the chamber, and the animals were once again allowed to explore both sides of the chamber for 20 min (1,200 s); the time spent on the side of the chamber associated with the test compound was determined. Preference score (in %) was calculated as 100 × [(test − baseline)/(600)].

### 2.10. CPP/CPA after subchronic EPD1504 or buprenorphine

The baseline time spent on either side of the CPP chamber was determined during the pretest as described in the above sections. Subsequently, the animals were implanted s.c. with a 2ML1 osmotic minipump (Durect Corporation, Cupertino, CA, USA) containing either EPD1504 or buprenorphine. The pumps were removed 5–6 days after implantation. After 24 h, the vehicle was injected s.c. in the morning and injected with the test compound in the afternoon. The injections and conditioning procedures were repeated 2× at 24 h intervals. Preference was determined 48 h later as mentioned in the above sections in the CPP procedure in opioid–naïve rats.

### 2.11. Scoring of somatic signs of withdrawal

Experimenters blinded to treatment conditions scored somatic signs using weighted scores as previously described (37, 38). During the session, animals were monitored during two 5-min epochs at 2 and 12 min after injection. The following behaviors were counted and assigned weighted scores based on the number of occurrences: wet dog shakes (<3 score = 2; >3 score = 4), and escape attempts (jumps with all 4 feet off the ground: 2-4,5-9 and >10 jumps were scored  1, 2, and 3, respectively). Any occurrence of teeth chattering/excessive facial grooming (score = 2), abdominal constriction (abdominal twitches; score = 2), pronounced swallowing movements (score = 2), abnormal posture (indicative of visceral discomfort; score = 3), ptosis (score = 2), erection or ejaculation (score = 3), chromodacryorrhea (porphyrin on the face; score = 5), profuse salivation (score = 7), vocalization on handling (score = 3). After the session, fecal pellets and/or diarrhea were counted and scores assigned (pellets >15/diarrhea; score = 2), and any weight change (score = 1 per 1% loss). A global weighted score was then calculated per animal per session.

### 2.12. Open-field test

Individual animals were injected with test compound 20 min prior to being placed in an open-field arena: a custom-built plexiglass chamber 38′′ × 38′′ × 18′′ height that was further enclosed in a sound-attenuating box purchased from Med Associates, Inc. (Fairfax, VT, USA). Animals were tracked using the videography from the camera positioned above the chamber using the Viewer2 software (Biobserve GmbH).

### 2.13. Pharmacokinetics: collection and analysis of plasma samples

To determine plasma exposures in the animals implanted with ALZET minipumps: the animals were lightly anesthetized with isoflurane, approximately 0.75 mL of blood was collected from a tail vein and mixed with 0.1 M EDTA. Samples were centrifuged at 4°C for 15 min. The supernatant was stored at −80°C until the analysis was carried out. The samples were analyzed by LC–MS at Quintara Discovery (Hayward, CA, USA). The pharmacokinetics (PK) of EPD1504 after acute injection were determined using cannulated rats at BioDuro, Inc. (San Diego, CA, USA) using a similar methodology.

### 2.14. Whole-body plethysmography

Whole-body plethysmograph chambers, hardware, and recording software from DSI (St. Paul, MT) were used to record unrestrained and unanesthetized respiration, with a negative bias flow of 2.5 L/min. Chambers were enclosed in a sound attenuating box purchased from Med Associates, Inc. (Fairfax, VT, USA). Animals were acclimatized to the chamber for 3 days, 40 min/day. After acclimatization, and for experiments in normal air, the animals were placed in the chamber and recorded for 30 min; subsequently, the chambers were opened and the animals were injected with the test compound. Then the animals were returned to their respective chambers, and their respiration was recorded for 1–2 h.

For experiments to monitor the response to 10% CO_2_, i.e., the hypercapnic ventilatory response (HCVR), animals were injected with the test compound 20 min before being placed in the chamber, and respiration was recorded for 30 min. Then, the negative bias flow was switched off, and a hypercapnic gas mixture of 10% CO_2_, 20% O_2_, and 70% N_2_ was fed into the chamber at approximately 2 psi. After 2 min, the bias flow was restarted, next the hypercapnic gas mixture was switched off. The data were normalized by dividing by the average minute volume observed during the last 15 min of the initial 30-min period.

### 2.15. Experimental design and statistical analysis

Statistics were performed using Prism (GraphPad Software, Inc., San Diego, CA, USA). Unless otherwise stated, for between subjects’ comparison, the data were analyzed using one-way analysis of variance (ANOVA) and Dunnett’s multiple comparisons *post*-*hoc t*-test, and are presented as mean ± standard error of measurement (SEM).

## 3. Results

### 3.1. EPD1504 is a brain-penetrant MOR partial agonist with buprenorphine-like antinociceptive activity

#### 3.1.1. EPD1504 is a MOR partial agonist

In CHO cells, stably expressing the human MOR, EPD1504 and buprenorphine exhibited similar limited (partial) efficacy compared to the high efficacy MOR agonist DAMGO (*E*_max_ ± standard deviation [SD] as percent response of DAMGO: EPD1504 56 ± 2%, and buprenorphine 63 ± 2%) (Figure 1B). In the Eurofins DiscoverX 78 safety scan, EPD1504 exhibited good selectivity and high affinity for the human MOR (0.0006 μM); potential off-target interactions that may occur based on *in vitro* affinities <10 μM were detected at targets, including δ (0.04 μM)- and κ (0.3 μM)-opioid receptors, and the dopamine D2 receptor (0.1 μM), i.e., at 67×, 500×, and 167× lower affinities than for the MOR. Additional potential off-target interactions detected between >2 μM and < 10 μM were observed at Nav1.5 (2 μM), SERT (3 μM), and nuclear hormone receptor (NR3C1) (9 μM). As detailed in the next section, 10 mg/kg s.c. achieves a plasma concentration of approximately 0.3 μM. Therefore, these off-target interactions unlikely occur in the dose range tested (<3 mg/kg s.c.).

#### 3.1.2. EPD1504 pharmacokinetics, MORs binding and antinociceptive activity in the hotplate test

At 1 h after 10 mg/kg s.c. was injected into rats (*n* = 3), EPD1504 was detected in plasma (171 ± 35 ng/mL) and cerebrospinal fluid (17 ± 9 ng/mL) (mean ± SD). The molecular weight of EPD1504 is 395 g/mol; therefore, 10 mg/kg s.c. achieves a plasma exposure of 171 ng/mL or 0.3 μM, and occupancy of >80% of CNS MORs; the half-life of EPD1504 was estimated to be 1.5 h. To confirm that EPD1504 binds and activates MOR in the CNS, we used a modified hotplate assay and receptor occupancy as described in our previous study (35). In the hotplate test, two doses of EPD1504 (1 and 10 mg/kg s.c.) and buprenorphine (0.03 and 0.3 mg/kg s.c.) that occupied approximately (20 and > 80%) of CNS MORs exhibited dose-dependent antinociceptive activity (Figures 1C-F). The data used to calculate CNS MOR occupancy (in %) for buprenorphine were reported in our previous study (35). Importantly although naloxone blocked the antinociceptive activity of both compounds (EPD1504 10 mg/g (79.6% vs. 13.9%, *p* < 0.001) and buprenorphine (84.5% vs. 1.9%, *p* < 0.001)), naloxone methiodide (39, 40), which does not cross the blood–brain barrier, had no effect on the antinociceptive effect of either compound (EPD1504 10 mg/kg + naloxone methiodide and buprenorphine 0.3 mg/kg + naloxone methiodide: 67.5 and 70.2%, respectively) (Figures 1E,F).

### 3.2. EPD1504 ameliorates OCD-like behaviors with reduced motor impairment

#### 3.2.1. EPD1504 and buprenorphine reduce error responses in a probabilistic reversal task

A schematic of the task is shown in Figure 2A. Briefly, a food-restricted rat can press either lever in the operant chamber to obtain a food reward. The probability of a reward after pressing the “correct lever” is 80%, whereas the probability of a reward after pressing the “error lever” is 20%. After eight correct-lever presses, the contingencies reverse, i.e., the correct lever becomes the error lever and vice versa.

The first reversal of each daily session was not included in the analysis. The data for 62 rats during their three baseline sessions (1,410 reversals) are presented in Figure 2B. During approximately 60% of baseline reversals, there were more correct responses than error responses (853 reversals in which correct > error, and 557 reversals in which error > correct). This result indicated that the rats had learned the contingencies of the task. The distribution of correct and error responses was not a normal distribution (Kolmogorov–Smirnov test: distance 0.1, *p* < 0.0001). Therefore, data were normalized to the interquartile confidence intervals (CIs) of baseline responses using the equation: (Probability of an error response during a reversal −25% CI)/(75% CI − 25% CI) as reported previously (41). As a measure of motor function, we monitored the rate of lever pressing (correct + errors), as shown in Figure 2C. The rates of lever pressing were stable over the 3-day baseline period.

Injection of vehicle did not affect either the lever-pressing rate (correct + errors presses per hour) or error responses per reversal (Figures 2C,E). Of the two serotonin modulators tested (mCPP and fluoxetine), only fluoxetine dose-dependently decreased error responses, without reducing the total lever-pressing rate. In contrast, although mCPP dose-dependently reduced the rate of lever pressing, it did not modulate the fraction of error responses per reversal (Figures 2D,E).

Of the three MOR ligands tested, doses of EPD1504 that occupied less than 60% of CNS MORs (Figure 2D) reduced the probability of an error response without impairing the lever pressing. While buprenorphine improved the performance, impaired lever pressing was observed at 0.03 mg/kg, a dose that occupies 20% of CNS MORs, i.e., buprenorphine reduced lever pressing at a lower level of CNS MORs occupancy compared to EPD1504. The MOR antagonist, naloxone, did not affect either lever-pressing rate or task performance (Figure 2E).

#### 3.2.2. EPD1504 and buprenorphine reduce marble burying

To improve consistency between experimenters, we developed a semi-automated method to analyze the number of marbles buried. In this analysis, the visible area of each marble was normalized by dividing by the mean marble area visible before the rat was placed in the chamber. Marbles that had a normalized area < 0.35 were classified as buried. No marbles were classified as buried before the animal was placed into the chamber. The mean normalized area and 5–95% CIs for 1,155 marbles before the rats were placed in the cage were (1 and 0.87–1.16), respectively (Figures 3A–D).

Fluoxetine dose-dependently reduced the number of marbles buried (Figure 3E). mCPP was not tested as it impaired lever-pressing rates in the probabilistic reversal test (Figure 2D).

Doses of EPD1504 1 mg/kg and buprenorphine 0.01 mg/kg that both reduced marble burying and increased the fraction of correct responses in the probabilistic reversal task, that improved performance in the probabilistic task without impairing the rate of lever pressing (as shown in Figure 2) reduced the number of marbles buried to the same extent (test compound dose [mg/kg] [marbles buried mean ± SEM]: EPD1504 1 mg/kg (3.1 ± 0.7) vs. buprenorphine 0.01 mg/kg [3.1 ± 1.1]) (Figure 3E); it is important to note that multiple comparisons revealed that no further decrease in marble burying was observed at higher doses that reduced lever-pressing rates in the probabilistic task: EPD1504 (1 mg/kg [3.1 ± 0.7] vs. 3 mg/kg [3.4 ± 1.0.8]), buprenorphine (0.01 mg/kg [3.1 ± 1.1] vs. 1 mg/kg [1.4 ± 0.7]), and EPD1504 (1 mg/kg vs. buprenorphine 0.03 mg/kg [*p* = 0.7]). Although a trend was noted, naloxone did not reduce the number of marbles buried (Figure 3E).

Doses of EPD1504 1 mg/kg and buprenorphine 0.01 mg/kg did not impair locomotor activity in the open-field test (Figure 3F). Finally, preinjection with naloxone blocked the effects of EPD1504 and buprenorphine, indicating that the reduction in marble burying is attributable to opioid receptors (Figure 3G).

### 3.3. EPD1504 has limited effects on respiration

We investigated the effects of EPD1504 on respiration using whole-body plethysmography in unrestrained animals under three conditions: (1) in normal room air (normoxic conditions), (2) with coinjection of diazepam at 3 mg/kg [a dose that has been shown to induce place preference in rats (42)], and (3) in elevated CO_2_ (10% CO_2_ hypercapnic conditions).

**Figure 4:**
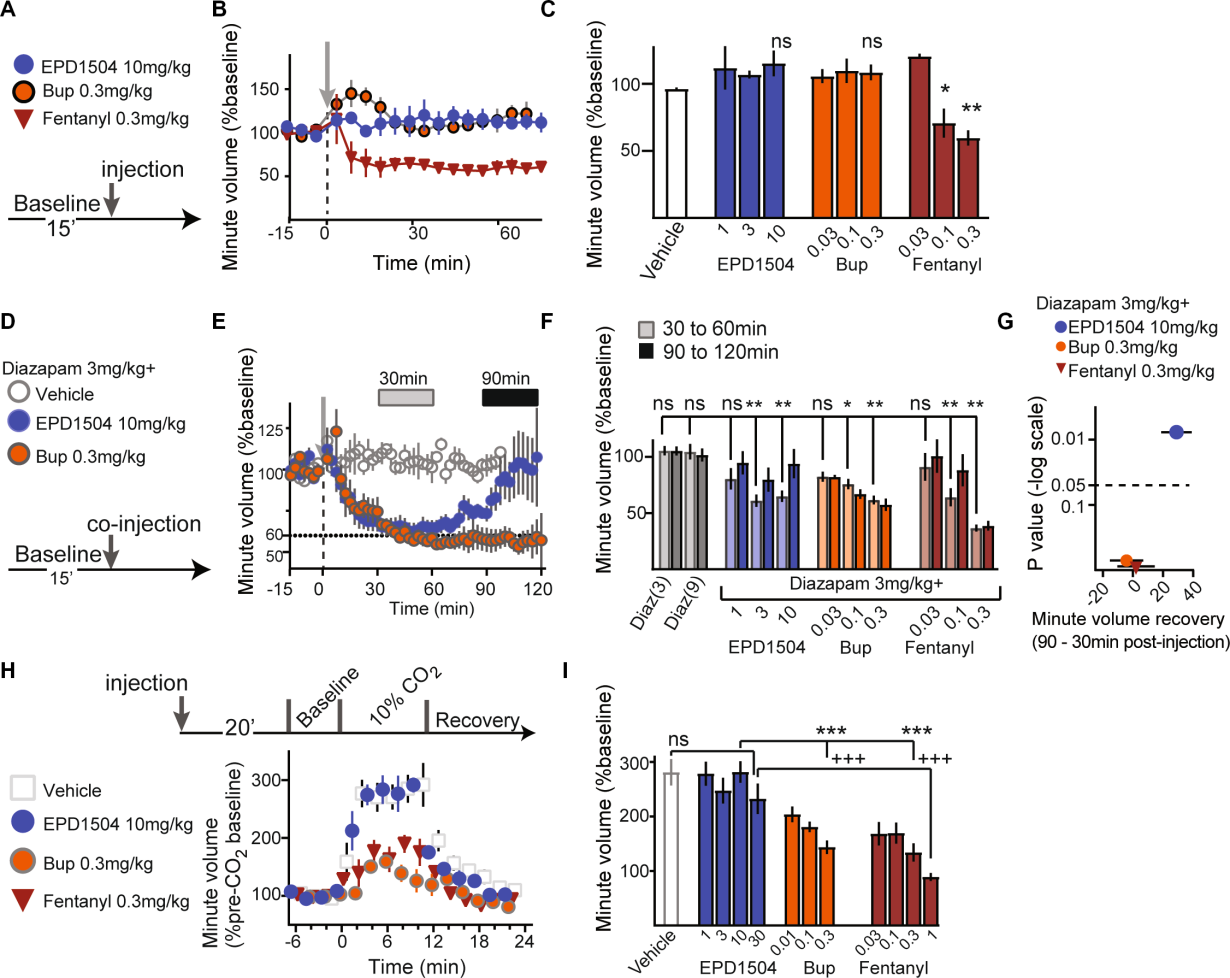
EPD1504 has limited effects on respiration. **(A–C)** Timeline, and summary data (mean minute volume between 30 and 60 min after injection) for effect of test compounds on minute volume under normoxic conditions (normal room air). The doses are listed in mg/kg below the *x*-axis; note that in contrast to EDP1504 and buprenorphine, fentanyl suppressed respiration compared to vehicle. **(D, E)** Timeline and data with inset bars at 30 and 90 min after injection that correspond to observed respiratory depression and recovery, respectively after coinjection of diazepam with test compounds. **(F)** Summary data for effect of coinjection of diazepam with test compounds on respiration at the two time points (30 and 90 min) illustrated in **(E)**. **(G)** Volcano plot of differences between mean minute volume observed at 30 and 90 min (recovery – depression); note that although coinjection of diazepam with all opioids tested exacerbated respiratory depression in normoxic condition, that only EPD1504 exhibited significant recovery to baseline in the at the 90-min time point. **(H,I)** Timeline and summary data for effect of test compounds on minute volume in 10% CO_2_ (hypercapnic conditions); note that unlike both buprenorphine and fentanyl, EPD1504 did not suppress respiration under hypercapnic conditions (All groups *n* = 5–6, *p < 0.05, ***p <0.001, and +++*p* <0.001, **p < 0.01 as indicated; ANOVA *post-hoc* test).

In normal room air, EPD1504 and buprenorphine had limited effects on respiration, whereas fentanyl (0.3 mg/kg) significantly suppressed respiration (Figures 4A–C). When coinjected with diazepam, all tested compounds initially (between 30 min and approximately 60 min after coinjection) caused a 30–40% decrease in respiration compared to animals injected with diazepam only. In rats coinjected with diazepam and EPD1504, respiration recovered within the 2-h recording period (90–120 min). In contrast, at the same time point, no recovery was observed after the coinjection of diazepam with buprenorphine. Analysis of differences between mean recovery (90 min) and initial depression (30 min) revealed that only EPD1504 exhibited a significant recovery. It is important to note that diazepam (9 mg/kg, i.e., at 3× the dose was coinjected with the test compounds) did not suppress respiration (Figures 4D–G). Under hypercapnic conditions, EPD1504 did not affect the response to 10% CO_2_, whereas buprenorphine and fentanyl dose-dependently suppressed the hypercapnic ventilatory response to similar extents (Figures 4H,I).

### 3.4. EPD1504 has a limited dependence liability

**Figure 5:**
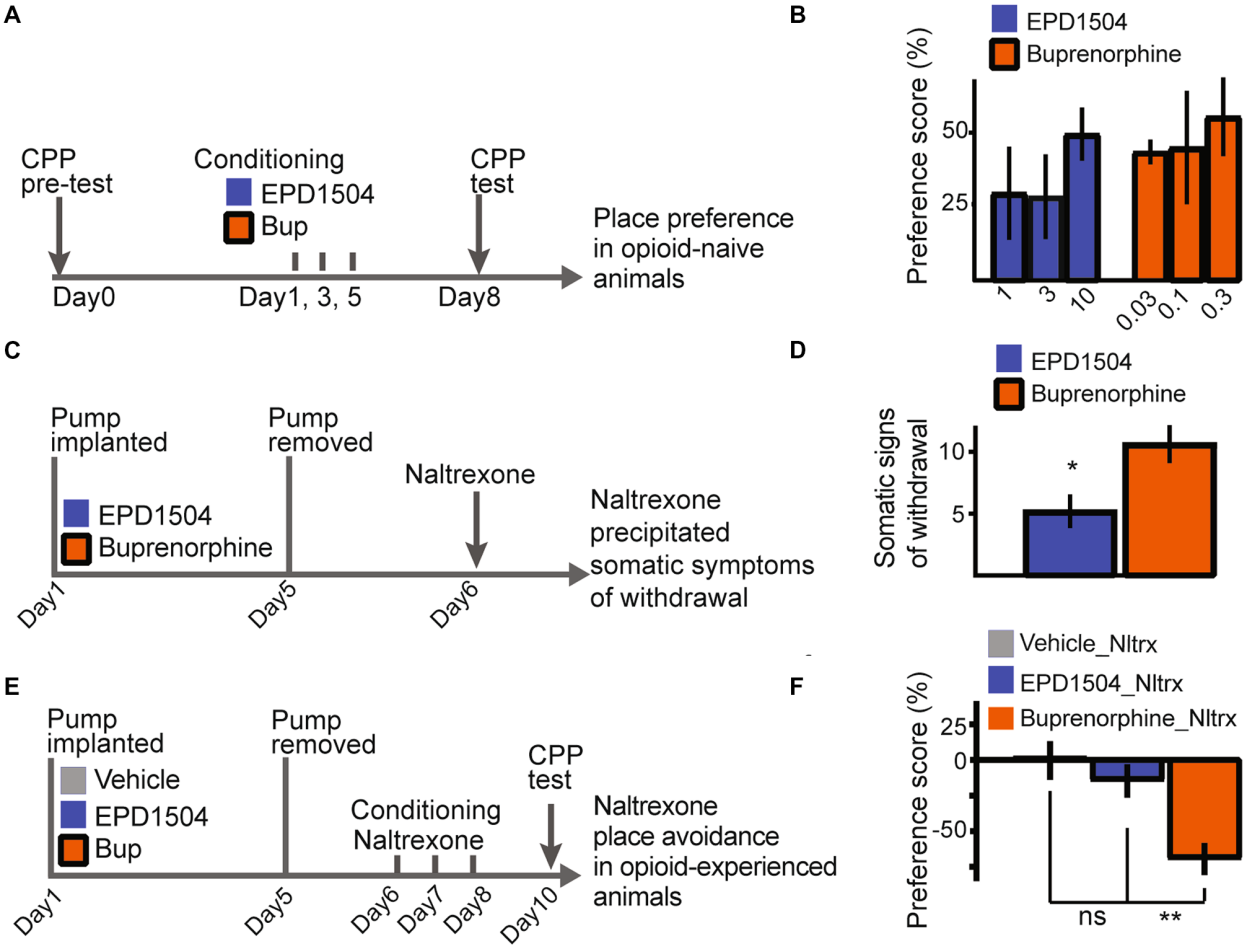
EPD1504 has a limited dependence liability. Timelines and summary data: in **(A,B)** for conditioned place preference observed in opioid–naïve rats in **(C,D)** for somatic signs of withdrawal precipitated by naltrexone following subchronic exposure to EPD1504 and buprenorphine in subcutaneous mini pumps. Note that as detailed in the main text plasma levels observed for both compounds achieve approximately 70% occupancy of CNS MORs. In **(E,F)** for conditioned **(E,F)** place aversion observed after injections of naltrexone (Nltrx) (All groups *n* = 6–11, ns = not significant, *p < 0.05, **p < 0.01 as indicated; ANOVA *post-hoc* test).

In opioid–naïve rats, EPD1504 and buprenorphine induced comparable levels of place preference (Figures 5A,B). As described in our previous study, naltrexone precipitated somatic signs of withdrawal and place aversion were used to investigate the dependence liabilities of EPD1504 compared to buprenorphine (35). Subcutaneous pumps (continuous infusion at 10 μL/h) that achieved comparable levels of occupancy of CNS MORs (approximately 70%): EPD1504 (5 mg/mL) 68 ± 6% (*n* = 4) and as reported in our previous study for buprenorphine (1 mg/mL) (35), were removed after 5 days. After this subchronic exposure, naltrexone (0.3 mg/kg) induced fewer somatic signs of withdrawal and conditioned place aversion in animals exposed to EPD1504 (Figures 5C–F).

## 4. Discussion

### 4.1. Evidence for opioids in OCD

MORs modulate both behaviors (e.g., behavioral flexibility) and underlying cortico–striatal circuits that are disrupted in OCD (43–46). In addition, opioids modulate neurotransmitters implicated in OCD, e.g., serotonin (47) and glutamate (48). These mechanistic observations motivated a series of investigator-led trials in treatment-resistant OCD patients; in a subset of these patients MOR agonists ameliorated OCD symptomology (4, 6, 8–10), whereas antagonists in many cases exacerbated symptoms (7, 11, 49, 50).

Two of the compounds used in the aforementioned OCD clinical trials (the partial agonist: buprenorphine and the antagonist: naltrexone) are U.S. Food and Drug Administration (FDA)-approved treatments for opioid use disorder, a population in which OCD is approximately 10× more prevalent than the general population (51–57). Evidence from the opioid use disorder population strongly supports the postulate that opioid agonists ameliorate OCD symptoms. First, OCD symptoms precede opioid misuse in approximately 70% of cases in which opioid use disorder and OCD are comorbid; implying that opioid misuse may be an effort to self-medicate for OCD (58, 59). Second, OCD symptoms are bi-directionally regulated during treatment of opioid use disorder: initially, a reduction in symptoms is observed during induction into treatment when patients receive higher doses of MOR agonists, whereas an exacerbation is observed upon agonist titration (60–64).

### 4.2. Factors regulating the therapeutic efficacy and liabilities of MOR agonists in OCD

The therapeutic effects and liabilities (sedation, reward, dependence, and respiratory depressant effects) of MOR ligands are primarily attributable to a combination of the following 3 factors: (1) how the ligand activates the MOR and the intracellular second messengers that are recruited, (2) the percentage of MORs bound in CNS circuits, and (3) interactions with active metabolites and other CNS active ligands, e.g., benzodiazepines.

#### 4.2.1. How the ligand activates the MOR

EPD1504 and buprenorphine are relatively selective partial agonists of the MOR. Most partial MOR agonists also exhibit limited activation of the second messenger Beta-Arrestin; although, it is an area of active research, limited intrinsic MOR efficacy and B-Arrestin signaling have been proposed to underlie the reduced liabilities of MOR partial agonists (27, 65).

Compared to other partial agonists (with comparable or lower intrinsic MOR efficacy), EPD1504 (like buprenorphine) is somewhat unique in that it exhibits limited off-target agonist activity; examples of other partial MOR agonists with off-target agonist activity include nalbuphine and butorphanol that are both potent MOR and kappa opioid receptor agonists, the latter has been shown to induce hallucinations and dysphoric effects (66, 67). Other examples include the prodrug tramadol and tapentadol, which are both partial MOR agonists and potent amine modulators, importantly the metabolites of tramadol are high-efficacy MOR agonists (68, 69). Our experiments confirmed that the limited activation of MORs (by both EPD1504 and buprenorphine) was sufficient to reduce OCD-like behaviors in 2 rat models, and that the MOR antagonist naloxone blocked the effects of both partial agonists in the hotplate and marble-burying assays.

#### 4.2.2. The percentage of MORs bound in the CNS

To investigate dose-dependent occupancy of CNS MORs by EPD1504 and buprenorphine, we used two assays; the first was a modified hotplate assay previously shown to provide an estimate of CNS MOR occupancy (35). In this assay, naloxone methiodide, a naloxone derivative that does not cross the blood–brain barrier (39, 40, 70, 71), did not affect the antinociceptive effects of either compound. In contrast, naloxone (which crosses the blood–brain barrier) antagonized the antinociceptive effects of both compounds. In the second experiment, we determined the fraction of carfentanil binding to MORs 1 h after injecting the test compounds. Doses of both molecules that occupied less than 20% of CNS MORs modulated behavior in both models of OCD-like behavior (probabilistic reversal task and marble burying assays). Critically, EPD1504 exhibited a much larger therapeutic window compared to buprenorphine, wherein EPD1504 impaired lever pressing at approximately 60% occupancy of CNS MORs, whereas at 20% occupancy of CNS MORs, buprenorphine impaired both lever pressing and respiration (the hypercapnic ventilatory response to CO_2_).

Our observations that low doses of buprenorphine reduce OCD-like behaviors in rat models, but that buprenorphine has a limited therapeutic window (limited separation between therapeutic and adverse events) is in agreement with clinical results. In clinical studies, low doses of buprenorphine (0.2–4 mg) ameliorate OCD symptoms (4, 9); [for comparison, the approved daily dose for sublingual buprenorphine in opioid use disorder treatment is significantly higher: 8–24 mg (72)]. Although the bioavailability and pharmacokinetics of buprenorphine are highly variable, pharmacokinetic models that predict dose-dependent plasma exposure and percent occupancy of CNS MORs have been developed (30, 73). From these clinical studies, 0.6 mg i.v. and 4 mg sublingual achieve a steady state plasma concentration of approximately 1–2 ng/mL; 1 ng/mL is estimated to occupy approximately 50% of CNS MORs in heroin-dependent volunteers (30). Therefore, the dose range used in the aforementioned OCD trials (0.2–4 mg) is projected to achieve a maximum plasma concentration of 1 ng/mL and to occupy approximately 2.5 to 50% of CNS MORs; caveats to this calculation include comparison of doses across studies and differences in MOR availability between healthy volunteers vs. opioid-dependent volunteers (74). Despite these caveats, it is interesting to note that in healthy volunteers, doses in (and below) this range induce dizziness and impair both cognition and balance; the dose range reported in previous studies is 0.075–0.6 mg i.v. (75–78). This body of clinical work on buprenorphine implies that there is a narrow dose range (and corresponding narrow CNS MOR occupancy range) in which buprenorphine ameliorates OCD symptoms without adverse events (including sedation and impaired cognition).

Given that both molecules (EPD1504 and buprenorphine) have similar *in vitro* properties and, as mentioned naloxone sensitive antinociceptive activity in the hotplate test, potential explanations for the larger therapeutic window of EPD1504 include off-target binding of buprenorphine that is sufficient to regulate its analgesic effects (69, 79), and as discussed in the following sections, the three active metabolites of buprenorphine.

#### 4.2.3. Interactions with active metabolites and other CNS active ligands, e.g., benzodiazepines

Compared to both buprenorphine and morphine, the *in vivo* effects of EPD1504 should be more predictable because they can be attributed to the intrinsic MOR efficacy of the parent compound; conversely, the results of both legacy molecules (buprenorphine and morphine) are less predictable as they can be attributed to a combination of off-target effects of the parent compound and/or its metabolites, i.e., buprenorphine and its higher efficacy metabolites including norbuprenorphine (31, 80–84) and morphine and its metabolite M6G (85, 86).

In a clinical study (85), morphine was compared to oliceridine: more predictable pharmacodynamics were observed with oliceridine (a MOR agonist which does not have an active metabolite); in contrast, a dissociation between the antinociceptive and respiratory depressant effects of morphine was observed and attributed to its active metabolite M6G. Our results comparing EPD1504 to buprenorphine are comparable: both compounds produced similar levels of anti-nociception in the hot plate test (during the 4-h period after injection as shown in Figure 1), but buprenorphine inhibited the respiratory response to CO_2_ and had prolonged respiratory depressant effects when combined with diazepam. Although the disparity in kinetics between EPD1504 and buprenorphine can in part be attributed to the difference in half-lives of the molecules (EDP1504: 1.5 h vs. buprenorphine 5 h) (87), we also observed that naltrexone precipitated fewer somatic signs of withdrawal and place aversion in rats after 5 days of continuous subchronic exposure to EPD1504; these results indicate that a higher level of dependency had developed for buprenorphine. As mentioned, given that the molecules have similar intrinsic efficacy at the MOR and that the ALZET minipumps provide continuous exposure, the observed differences are likely due to the higher efficacy metabolites of buprenorphine (35). Therefore these data indicate that EPD1504 will have more predictable pharmacokinetic and pharmacodynamic effects. As observed in our studies, this led to a broader therapeutic window in which EPD1504 could be dosed up to approximately 60% occupancy of CNS MORs without adverse effects being observed.

### 4.3. OCD models used, pharmacology in OCD models used, and limitations

#### 4.3.1. OCD models used

The validity of OCD behavioral models (face, predictive, and construct validity) has been reviewed extensively (13, 22, 88). Concisely, face validity gauges how well the animal’s behavior recapitulates pathological human behavior. If pharmacological responses or circuit pathologies of the model are comparable to those observed in patients, then it is considered to have good predictive and construct validity. As mentioned in the introduction, the probabilistic reversal task can be applied to humans and rodents, and task performance is disrupted in OCD patients (14). Additionally, first-line OCD approaches, including serotoninergic manipulations, regulate performance in healthy volunteers (15, 16) and in rodents (17, 18).

Marble burying in rodents is proposed to model compulsions observed in OCD patients. Pharmacological agents that reduce marble burying without reducing locomotor activity in the open-field test are predicted to be anxiolytic or to ameliorate OCD-like compulsions without inducing significant sedation or motor impairment (19–23).

#### 4.3.2. Pharmacology in the OCD models used

In keeping with previous work reviewed by (20, 21), we observed that the SSRI, fluoxetine, ameliorated OCD-like behaviors in both models. In contrast, mCPP: a 5HT_2C_-preferring agonist that also binds serotonin transporters that dose-dependently exacerbates both rodent marble burying and OCD symptoms in patients (20, 89), impaired lever pressing in the probabilistic reversal task. These results with fluoxetine and mCPP indicate that the rate of lever pressing measured sedation and/or motor impairment.

In mice, acute opioids increase locomotor activity and decrease marble burying; in contrast, in rats acute opioids reduce locomotor activity (23, 90). Despite the opposing effects on locomotion (in rats and mice), we observed that the MOR agonists (EPD1504 and buprenorphine) reduced marble-burying at doses that did not impair locomotor activity. A similar dose-dependent separation between amelioration of OCD-like behavior and sedation (reduction in forward locomotion) was observed for buprenorphine in a model of OCD induced by a 5HT_2A_ agonist (91). In the probabilistic reveral task, opiate users being treated with the MOR agonist methadone are significantly slower than both healthy volunteers and other substance use disorder patients (92). Furthermore, ketamine modulates performance on the task in rats (18) as well as ameliorates OCD symptoms in patients (93). Although ketamine is primarily considered an NMDA receptor antagonist, its effects are blocked by MOR antagonists in patients and rodents suggesting downstream activation of MORs (94, 95).

#### 4.3.3. Limitations of OCD models used

OCD is often comorbid with other psychiatric indications. Depending on etiology (genetic predispositions, comorbidities, or endophenotypes, e.g., alcoholism, or ticks), MOR antagonists (rather than agonists) will likely be more appropriate therapeutics (58, 96, 97). In our studies using wild-type animals, the MOR antagonist (naloxone) did not affect behavior in the two models tested. Therefore, future studies using genetic or pharmacological models will be needed to investigate the role of MOR antagonists in distinct subsets of OCD-like behaviors (98). Another limitation beyond the scope of the current study is that we did not seek to correlate observed behavioral effects and levels of CNS MOR occupancy with either biomarkers associated with OCD (99) or with potential mechanisms, e.g., neuronal circuit function.

### 4.4. Safety

The therapeutic efficacy of MOR agonists must be balanced with the associated risks of abuse and respiratory depression. Although buprenorphine and tramadol have lower abuse liabilities compared to full efficacy MOR agonists, they are abused (or misused to self-medicate) in populations with restricted access to opioids (25, 82, 100). In preclinical studies, both buprenorphine and tramadol induce place preference (as reviewed in (101)). In our studies, EPD1504 and buprenorphine induced comparable levels of place preference indicating comparable reward liabilities, but EPD1504 induced significantly less dependence as measured by naltrexone-induced somatic signs of withdrawal and place aversion (35, 102). Compared to buprenorphine, EPD1504 had reduced respiratory liabilities at all doses tested. These findings are consistent with the effects of other agonists with lower intrinsic MOR efficacy based on the same molecular scaffold as EPD1504 (35). Although future studies will be needed to confirm these results, our results indicate that EPD1504 should have reduced respiratory and dependence liabilities compared to buprenorphine.

### 4.5. Summary

OCD affects a substantial fraction of the population (2–3%) and is associated with high rates of disability (1, 103, 104). Current first-line treatments have a slow rate of onset and are ineffective in approximately half of OCD patients. In a subset of these treatment-resistant patients, low doses of MOR agonists rapidly ameliorate OCD symptoms; e.g., (4, 8, 10); notably, MOR agonists have a long history of efficacy in psychiatry conditions (48, 105). Notwithstanding the complex etiology of psychiatric disorders (including OCD), preclinical MOR pharmacology and pharmacokinetic translate surprisingly well to clinical studies; in our studies, despite the many caveats, levels of occupancy of CNS MORs correlated well with the levels of occupancy in human studies that, respectively, provide therapeutic effects, and sedation. In addition, as discussed above, the probabilistic reversal task has provided translatable insights into the pharmacology and circuit basis of OCD pathology.

Despite solid clinical and supporting translational evidence, the utility of MOR agonists is limited by their inherent dependence, abuse, and respiratory liabilities. The improvements offered by EPD1504 and like molecules on these liabilities (i.e., reduced respiratory effects and dependence), and more predictable pharmacokinetics and pharmacodynamics, coupled with recently developed abuse deterrent approaches (e.g., slow-release subcutaneous implants) should dramatically improve the utility of opioids in psychiatric indications.

#### 4.5.1. Additional requirements

For additional requirements for specific article types and further information please refer to “Article types” on every Frontiers journal page.

## Data availability statement

The original contributions presented in the study are included in the article/supplementary material, further inquiries can be directed to the corresponding author.

## Ethics statement

The experiments involving animals were approved by the Life Source Biomedical Services Institutional Animal Care and Use Committee (IACUC) in accordance with the animal care standards set forth by the Office of Laboratory Animal Welfare (OLAW), National Institutes of Health (NIH).

## Author contributions

BY and NS participated in the research design and conducted the experiments. JM designed EPD1504, led chemistry and helped interpret data. NS performed the data analysis. DG and NS contributed to the writing of the manuscript. All authors contributed to the article and approved the submitted version.

## Funding

Epiodyne has received funding from National Institute on Drug Abuse (NIDA).

## Conflict of interest

BY, DG, and NS are employees of and stockholders in Epiodyne, Inc. JM is an Epiodyne stockholder, and a stockholder and employee of R2M.

## Publisher’s note

All claims expressed in this article are solely those of the authors and do not necessarily represent those of their affiliated organizations, or those of the publisher, the editors and the reviewers. Any product that may be evaluated in this article, or claim that may be made by its manufacturer, is not guaranteed or endorsed by the publisher.
